# Fecal Microbiota Transplantation Research over the Past Decade: Current Status and Trends

**DOI:** 10.1155/2023/6981721

**Published:** 2023-01-09

**Authors:** Shaodong Hao, Shanshan Yang, Naiwei Zhang, Hongjie Cheng

**Affiliations:** ^1^Spleen-Stomach Department, Fangshan Hospital, Beijing University of Chinese Medicine, Beijing, China; ^2^Graduate School, Beijing University of Chinese Medicine, Beijing, China

## Abstract

**Background:**

Fecal microbiota transplantation (FMT) is a current research hotspot, with a surge in the output of publications over the past decade. This study dedicates to the exploration of the research status and highlights significant themes and future trends in FMT research with the aid of bibliometric analysis.

**Methods:**

FMT publications from 2012 to 2021 were retrieved on August 12, 2022, using the SCI-Expanded of Web of Science (WoS). The Bibliometrix in R program, Microsoft Office Excel, VOSviewer, and CiteSpace were utilized for bibliometrics and visual analysis, revealing the main publications, journals, countries, agencies, authors, and keywords distribution in FMT research.

**Results:**

There were 2,931 papers included. FMT research presented a growing trend from 2012 to 2021. The countries with the most publications and contributions in FMT area were China and the United States. The high-yield institutions were Harvard University, Udice French Research Universities, and the University of California System. The primary authors were Nieuwdorp Max, Allegretti Jessica R, and Kassam Zain. Frontiers in Microbiology and Science were the top-ranked journals in publications and total citations, respectively. The important topics primarily included FMT-related mechanisms and the usage of FMT in Clostridium difficile infection (CDI), inflammatory bowel disease (IBD), irritable bowel syndrome (IBS), metabolic disease, neurological disorders, and psychiatric disorders. Future research would primarily concentrate on neurological disorders, chemotherapy and immunotherapy for malignant tumors, and FMT-related consensus and guidelines.

**Conclusion:**

With the help of bibliometric analysis, we were able to obtain the understanding of the status and trends of global FMT-related research. The field of FMT is undergoing tremendous progress, and our findings can guide clinical researchers' and practitioners' future work in the rapidly evolving field of FMT.

## 1. Introduction

Fecal microbiota transplantation (FMT) means that the transfer of functional GM of healthy people into the intestine of patients to restore the balance of the patient's GM or rebuild the GM to study the causal relationship between GM and disease. FMT can even be traced back to ancient China [1]. In 1958, Eiseman et al. [2] first reported that four patients with severe pseudomembranous colitis recovered after fecal enema, which is the origin of modern FMT research. In 1989, Bennet and Brinkman reported [3] the first patient with ulcerative colitis (UC) treated with fecal enema. However, it was not until 2013 that the U.S. Food and Drug Administration classified human feces as a drug, and FMT was first included in the treatment guideline for CDI [4]. Since then, FMT research had developed rapidly. In the past decade, FMT-related research in recurrent CDI (rCDI) [5] and inflammatory bowel disease (IBD) [6, 7] has made significant progress. In addition, there are increasing applications of FMT in multiple other diseases, such as irritable bowel syndrome (IBS) [8], constipation [9], liver disease [10], cancer [11, 12], diabetes [13], metabolic syndrome (MS) [14], autism [15], and neurological disorders [16].

Bibliometrics is a quantitative approach that employs mathematical and statistical strategies to assess the features of publications, enabling researchers to outline a complex graph of the knowledge structure and the development of specific knowledge, and has obtained successful application in methodology research, scientific disciplines, science policy, and so on [17, 18]. Analysis of relevant literature through bibliometrics can help us quantitatively and objectively identify past and present research topics, quickly understand classic literature in a field, and analyze future development trends. Scholars have achieved many valuable research results using bibliometric methods [19–21]. There are fewer studies on historical citation networks, cluster analysis of common keywords, and prediction of future development directions. To study the status and trends of FMT research, we selected the FMT-related literature from 2012 to 2021 in this study. With the help of bibliometrics, we described the features of the journals, authors, agencies, and countries and identified highly cited papers, current hotspots, and future trends in FMT research, which provided a reference for relevant researchers.

## 2. Materials and Methods

### 2.1. Data Sources and Retrieval Strategy

WoS Core Collection (WoSCC) is an essential database for getting worldwide scholarly information that has a tight screening procedure and only includes key publications in various areas. The WoSCC's SCI-Expanded database, which has been extensively used in previous bibliometric research [22], contains the most reputable and well-known mainstream publications in natural science. Therefore, WoSCC's SCI-Expanded was selected as the data source.

**Figure 1:**
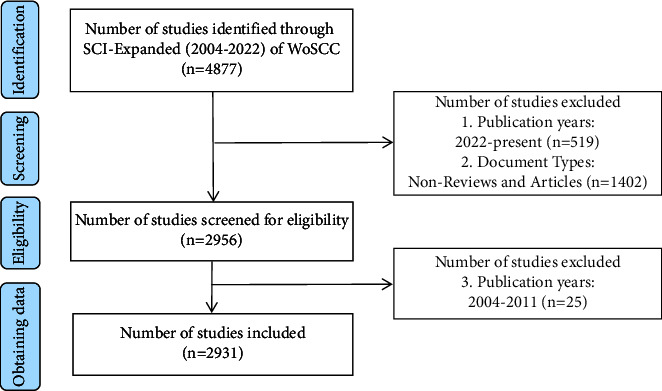
Diagram of paper search and screening process.

The retrieval strategy was Title (TI) OR Abstract (AB) OR Author Keywords (AK) = (“Fecal Microbi^*∗*^ Transplant^*∗*^” OR “Fecal Microbi^*∗*^ Transfer^*∗*^” OR “Fecal Microbi^*∗*^ Transplant^*∗*^” OR “Fecal Microbi^*∗*^ Transfer^*∗*^” OR “Intestinal Microbi^*∗*^ Transfer^*∗*^” OR “Intestinal Microbi^*∗*^ Transplant^*∗*^” OR “Gut Microbi^*∗*^ Transplant^*∗*^” OR “Gut Microbi^*∗*^ Transfer^*∗*^” OR “Fecal Microflora Transplant^*∗*^” OR “Fecal Microflora Transfer^*∗*^” OR “Fecal flora Transplant^*∗*^” OR “Fecal flora Transfer^*∗*^” OR “Fecal Microflora Transplant^*∗*^” OR “Fecal Microflora Transfer^*∗*^” OR “Fecal flora Transplant^*∗*^” OR “Fecal flora Transfer^*∗*^” OR “Intestinal Microflora Transplant^*∗*^” OR “Intestinal Microflora Transfer^*∗*^” OR “Intestinal flora Transplant^*∗*^” OR “Intestinal flora Transfer^*∗*^” OR “Gut Microflora Transplant^*∗*^” OR “Gut Microflora Transfer^*∗*^” OR “Gut flora Transplant^*∗*^” OR “Gut flora Transfer^*∗*^” OR “Fecal Transplant^*∗*^” OR “Fecal Transfer^*∗*^” OR “Fecal Transplant^*∗*^” OR “Fecal Transfer^*∗*^” OR “Donor Feces Infusion^*∗*^” OR “Feces Infusion^*∗*^ of Donor” OR “Infusion^*∗*^ of Donor Feces” OR “Fecal Bacteri^*∗*^ Transplant^*∗*^” OR “Fecal Bacteri^*∗*^ Transplant^*∗*^” OR “Gut Bacteri^*∗*^ Transplant^*∗*^” OR “Intestinal Bacteri^*∗*^ Transplant^*∗*^” OR “Enteric Bacteri^*∗*^ Transplant^*∗*^” OR “Transplant^*∗*^ of Fecal Microbi^*∗*^” OR “Transfer^*∗*^ of Fecal Microbi^*∗*^” OR “Transplant^*∗*^ of Fecal Microbi^*∗*^” OR “Transfer^*∗*^ of Fecal Microbi^*∗*^” OR “Transplant^*∗*^ of Gut Microbi^*∗*^” OR “Transfer^*∗*^ of Gut Microbi^*∗*^” OR “Transplant^*∗*^ of Intestinal Microbi^*∗*^” OR “Transfer^*∗*^ of Intestinal Microbi^*∗*^” OR “Fecal Bacteriotherap^*∗*^” OR “Fecal Bacteriotherap^*∗*^” OR “Fecal Enema” OR “Fecal Enema” OR “Stool Transplant^*∗*^” OR “Transplant^*∗*^ of Stool” OR “Stool substitute transplant^*∗*^” OR “Washed Microbi^*∗*^ Transplant^*∗*^”), search for synonyms according to the MeSH database from PubMed. The inclusion criteria include the following: (1) thesis types were limited to “article” and “review,” (2) publication years were from 2012 to 2021, and (3) there was no language limit. All searches were completed and downloaded on August 12, 2022. A total of 2391 FMT-related papers were finally obtained (Figure 1). We extracted the key information from the raw data and saved it in TXT format. These data do not relate to any personal information, so informed consent is not required. The impact factors (IFs) and JCR partitions refer to “Journal Citation Reports™ 2021.”

### 2.2. Data Analysis

The Bibliometrix R package (v 4.1.3 Windows, the R Foundation), VOSviewer (v 1.6.18, the Netherlands), CiteSpace (v 6.1.R3 Basic), and Office Excel 2019 (Microsoft, Washington, USA) were used in the analysis. The Bibliometrix contains a set of tools undertaking quantitative research in scientometrics [23]. VOSviewer is widely used for bibliometric analysis due to its more beautiful visualization, especially keyword cooccurrence analysis [24]. CiteSpace is a visualization analysis software gradually developed for scientometrics. To aid comprehension of FMT research, each program provides for the creation and display of bibliometric networks. These tools specifically examined the distribution of each analyzed component, including annual scientific output, most relevant sources or authors or affiliations, production and local impact of top journals and authors over time, country scientific production and collaboration network, historical citation network, high-cited papers and references, common keywords, and cluster analysis.

The number of papers (Np) can reflect the author's scientific productivity, and the analysis of the core authors can grasp the research trend and development trend of a certain field. However, relying solely on the Np does not evaluate an author's outstanding contribution to FMT research, through a series of scientometric indicators such as total citation (TC) and H-index can further evaluate the author's contribution. The TC is a useful index for determining the significance and effect of an author's cumulative papers and is used to assess a person's academic achievement. The H-index was developed by American physicist Jorge E Hirsch to assess individual academic achievements, and it was then applied to assess the academic influence of journals.

## 3. Results

From 2012 to 2021, total 2931 papers were obtained based on SCI-E of WoSCC. FMT-related papers were published in 875 journals by more than 15,000 authors from more than 3,000 institutions in 81 countries and regions, of which 1,940 were “articles” and 991 were “reviews.” The English literature accounted for 99.17% of the total Np.

### 3.1. Annual Papers Output

**Figure 2:**
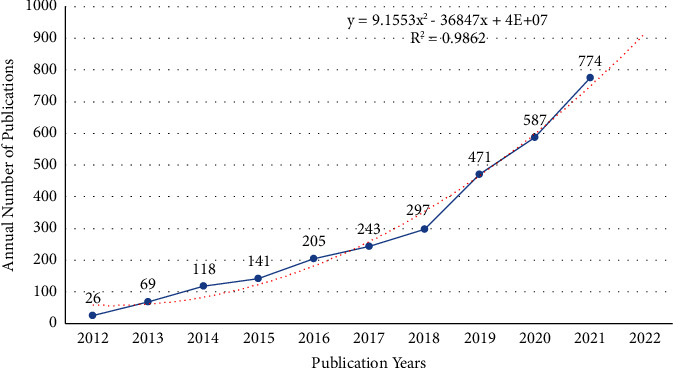
Annual papers output and fitting curve of publications in FMT.

Figure 2 shows that the Np in FMT research was on the rise from 2012 to 2021, and the annual average Np is 293. The Np increased slowly from 2012 to 2017. From 2018 to 2021, The Np rose rapidly before peaking in 2021 (*n* = 774, 26.4%). A polynomial model (*f(x)* *=* *p*_0_*x*^*n*^ *+* *p*_1_*x*^*n*−1^ + *p*_2_*x*^*n*−2^ + *p*_3_*x*^*n*−3^ + … + *p*_*n*_) was created to predict the output in 2022, and the formula was *y* = 9.1553*x*^2^ − 36847*x* + 4E + 07. The year and the Np showed a statistically significant link (*R*^2^ = 0.9862), and the goodness-of-fit was well. We predict that the Np on FMT would reach roughly 920 in 2022 based on the fitting curve.

### 3.2. Main Journals

**Figure 3:**
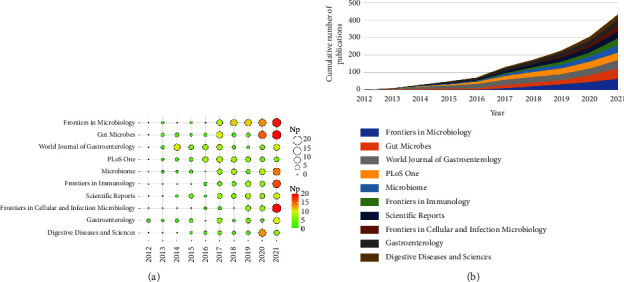
(a) Annual output of the top 10 journals in FMT. (b) Cumulative output of the top 10 journals in FMT.

Table 1 shows the academic output of top 10 journals. *Frontiers in Microbiology* ranked first in Np (*n* = 66), next were *Gut Microbes* (*n* = 62), *World Journal of Gastroenterology* (*n* = 52), *PloS One* (*n* = 45), and *Microbiome* (*n* = 44). The TC and H-index indicate the importance of the journals.  Table 2 lists the top 10 high-cited periodicals, among which *Science* (*n* = 9744) receiving the most citations, followed closely by *Gastroenterology* (*n* = 6522), *Gut* (*n* = 4339), *Microbiome* (*n* = 3361), and *Nature* (*n* = 3212). Moreover, *Microbiome* was at the top of H-index, followed by *World Journal of Gastroenterology*, *Gastroenterology*, *Gut*, *Gut Microbes*, and *PloS One* (Table 2). Figures 3(a) and 3(b) highlight the top 10 journals' yearly and cumulative output. These journals had the highest cumulative Np of 454, accounting for approximately 15.5% of total output, showing that they were the most prolific.

### 3.3. Major Countries and Institutions

Table 3 lists the top 10 high-yield nations and their TC and H-index, among which the United States (*n* = 1050) and China (*n* = 755) had the highest Np, with the ratio of about 61.6% of the total output, followed by Canada, Germany, and France. Moreover, the United States was at the top of TC and H-index, followed by China, France, Netherlands, and Canada. Table 3 also lists the top 10 high-yield institutions, of which Harvard University, Udice French Research Universities, University of California System, Inserm, and Harvard Medical School were among the top five. Also, Harvard University was at the head of Np and H-index, while Udice French Research Universities had the highest TC.

**Figure 4:**
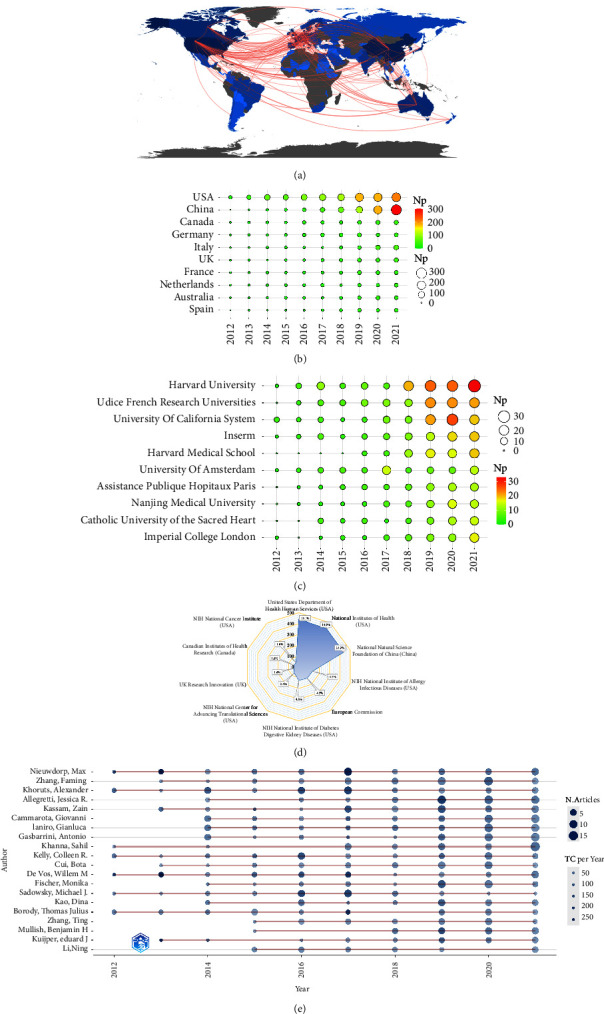
(a) Global scientific output and international cooperation network (the shade of color represents the national output; the more intense the blue, the greater scientific production. Grey shows countries without scientific production; the red line shows the cooperation, the thicker the boundaries, the greater the cooperation degree). (b) Annual output of the top 20 high-prolific countries in FMT (the circle's size manifests the national output; the bigger the circle, the more publications). (c) Annual output of the top 10 high-prolific institutions in FMT. (d) The top 10 funding organizations in FMT research. (e) Annual output of the top 20 most prolific writers in FMT research throughout time (the circle's size symbolizes the output, with larger circles representing more output; the circle's depth symbolizes the annual citations, with darker colors representing more citations).

Figure 4(a) lists the national scientific output and the international cooperation network. We can find that the international cooperation in FMT research was relatively close. The annual issuances by high-yield countries are shown in Figure 4(b). The Np in China was growing rapidly. In 2021, China surpassed the US to become the country with the largest Np.  Figure 4(c) shows the annual production of the top 10 high-yield agencies. The top 10 institutions produced articles every year between 2016 and 2021.  Figure 4(d) illustrates the major funding organizations, mostly from the United States and China, demonstrating that these countries are highly supportive of FMT-related studies.

### 3.4. Main Researchers

Table 4 ranks the top 10 high-yield authors (using the full name of the authors and their WoS researcher ID to reduce the repetition brought by name abbreviation), among which Nieuwdorp Max (*n* = 44), Zhang Faming (*n* = 44), Khoruts Alexander (*n* = 43), Allegretti Jessica R (*n* = 42), and Kassam Zain (*n* = 41) ranked the top five. Sorted by TC and H-index, it showed that in FMT research, the most influential authors were Nieuwdorp Max (7440, 27), Khoruts Alexander (4513, 27), Kelly Colleen R. (3246, 23), and Kassam Zain (3139, 23). The top 10 authors were from the US (*n* = 5), Italy (*n* = 3), China (*n* = 1), and the Netherlands (*n* = 1). Notably, the top 10 authors were from various institutions, showing broad interest in FMT.  Figure 4(e) lists the annual output of the top 20 authors. As we can see, the top 10 authors produced articles each year from 2017 to 2020. Notably, Allegretti Jessica R., Cammarota Giovanni, and Khanna Sahil had gradually increased their output in recent years and could publish more papers in the next few years.

### 3.5. Classic and High-Cited Papers

#### 3.5.1. Historical Cited Papers in FMT

**Figure 5:**
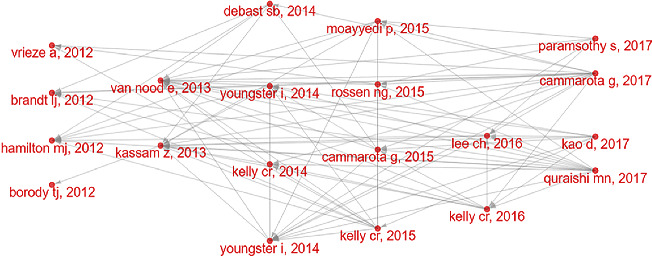
Historical FMT-related citation network (each dot displays a paper, the lines between dots highlight the links between studies, and the corresponding papers are shown in Table 5).

By the aid of the historically cited papers analysis in the Bibliometrix, some classic FMT-related papers were found (Figure 5). To examine their research significance, two indicators, LCS (local citation score in the current dataset) and GCS (global citation score in the WoSCC database), were used.

The classical papers were published in 2012–2017 (Figure 5 depicts the citation network). In 2012, a multicenter follow-up study [25] showed prolonged efficacy of colonoscopic FMT for rCDI. A clinical study [26] summarized standardized frozen preparation for FMT in rCDI treatment, marking the transition of FMT research to standardization. Moreover, a study found that after the infusion of microbes from lean donors, the insulin sensitivity of recipients increased, demonstrating the favorable effect of reconstituted GM on metabolic syndrome [27]. A review outlined the use of FMT in CDI and its promise in other GM dysfunction-related diseases [28]. In 2013, a paper, with the highest LCS and GCS, showed that infusion of donor stool was a potential therapeutic strategy for rCDI, which was the first controlled clinical study of FMT in treating rCDI-related diarrhea [5]. A meta-analysis further confirmed that FMT had good efficacy and safety in CDI [29]. In 2014, several randomized controlled trials (RCT) of FMT in CDI treatment were published [30–32], and the guideline for CDI treatment was updated, which strongly recommend FMT for multiple rCDI [33]. In 2015, a RCT further showed that FMT using colonoscopy-infused stool for rCDI was significantly superior to the vancomycin regimen [34]. Two clinical studies in *Gastroenterology* showed that FMT had good clinical efficacy and safety in individuals with ulcerative colitis (UC) [7, 35]. A review outlined the indications, methods, and mechanisms of FMT [36].

In 2016, a RCT confirmed that frozen FMT was comparable to fresh FMT in treating diarrhea in adults with rCDI, suggesting that frozen FMT has potential clinical advantages [37]. The other RCT in *JAMA* confirmed that FMT using donor stool by colonoscopy seemed safe and was more effective in preventing CDI flares than FMT with the patient's own feces [38]. In 2017, another RCT in *JAMA* comparing oral capsule versus colonoscopy FMT on rCDI confirmed that oral capsule was noninferior in preventing recurrent infection in adults with rCDI for colonoscopy FMT [39]. A meta-analysis showed that FMT was effective in recurrent and refractory CDI treatment, regardless of setup method or delivery route [40]. A well-designed RCT demonstrated that FMT can promote clinical remission and endoscopic amelioration in active UC and was linked with significant microbial alterations, which was a promising new treatment option for UC [6]. Moreover, European consensus on FMT-related clinical practice was published [41]. The classic papers on FMT mainly focused on CDI and application reports other than CDI continued to emerge.

#### 3.5.2. Top 20 High-Cited Papers in FMT

Highly cited papers refer to the most influential papers in the present dataset in Table 5. According to the ranking of TC, important papers can be quickly located. The more citations, the higher the academic value of the paper, and new discoveries and trends can be found from the paper. The papers with high TC are generally important discoveries or new interpretations, often reviews or significant original articles.

As shown in Table 6, FMT was mainly used in mechanism research and clinical research, mainly focusing on CDI, UC, MS, cardiovascular and cerebrovascular diseases, mental diseases, and cancer immunotherapy. (1) CDI: Surprisingly, in the top 20 most cited papers, only one is about FMT treatment of CDI. The research showed that FMT was a potential therapeutic strategy for rCDI [5]. (2) UC: Two clinical studies have shown that FMT can provide relief in patients with active UC [7, 35]. FMT from ACE2 mutant mice into germ-free (GF) mice could impart an increased propensity to develop severe colitis [42]. (3) MS: A 2012 study showed, after 6 weeks of infusion of lean donor microbiota, recipients had increased insulin sensitivity and butyrate-producing GM [27]. A 2015 study showed that jet lag-induced dysbiosis can promote glucose intolerance and obesity, which can be transferred to GF mice after FMT [43]. Two articles published in *Nature* confirmed that artificial sweeteners can cause glucose intolerance by modulating the GM [44], and dietary emulsifiers can affect the GM in mice to promote colitis and metabolic syndrome [45], which were confirmed by FMT experiments. (4) Cardiovascular and cerebrovascular diseases: It was observed that hypertension can be transferred through FMT from hypertensive human donors to GF mice, demonstrating the direct effect of GM on host blood pressure [46]. Antibiotic-induced GM changes can reduce ischemic brain damage in mice, an effect that can be transmitted through FMT [47]. (5) Mental diseases (MD): Colonization of the “depressed microbiota” from patients with major depressive disorder caused depression-like behaviors in GF mice compared to “healthy microbiota” colonization from healthy individuals [48]. A study showed that anhedonia and anxiety-like behaviors, as well as changes in tryptophan metabolism, were all generated in recipient animals by FMT from depressed patients into microbiota-depleted rats [49]. The other study showed that FMT can alter GM and alleviate gastrointestinal and autism symptoms [15]. (6) Cancer immunotherapy: In 2015 and 2018, four studies showed that the drug resistant to immune checkpoint inhibitors (ICIs) was related to GM, notable changes in the GM between ICI responders and nonresponders were also noted, and FMT can enhance the antitumor effect of ICIs [50–53].

#### 3.5.3. Most Local Cited References of FMT Research

Local cited references refer to the most cited references in the present dataset. According to the ranking of TC, important references in the field can be quickly located. Checking the references can trace the development history of FMT, so we can have a more comprehensive understanding of FMT.

As shown in Table 7, we mainly reviewed the FMT-related articles before 2012, and found that the research types were mainly case reports. In 1958, Eiseman et al. [2] reported that four patients with severe pseudomembranous colitis recovered after fecal bacterial transplantation, which was the origin of modern FMT research (refer to several FMT review articles). In 1981, a study showed that 16 patients with pseudomembranous enterocolitis received restoration of floral homeostasis by fecal enema [54]. In 1983, a case report in *Lancet* showed rectal infusion of homologous stool may cure recurrent *Clostridium difficile* enterocolitis [55]. In 1989, an article in *Lancet* showed that implantation of normal colonic flora treats UC [3]. The other article reported the effect of bacteriotherapy on six patients with chronic recurrent *Clostridium difficile* diarrhea (rCDI-related diarrhea) [56]. Moreover, altering the GM could be a potential treatment for altering IBD and IBS [57]. In 2000, an article reported on the recurrent *Clostridium difficile* diarrhea treatment by direct administration of donated feces via colonoscopy [58]. In 2003, a case series reviewed the medical records of 18 subjects receiving donor feces via a nasogastric tube for rCDI and found favorable outcomes [59]. A case report showed that colonic infusion of donor feces could reverse UC in certain patients [60]. In 2004–2011, there were multiple case reports showing the potential role of FMT in CDI, which gradually attracted the attention of researchers. Simultaneously, some basic studies had shown that FMT can be used to study the mechanism of GM in disease occurrence [61, 62]. Notably, FMT-related research had been in a slow development stage until 2012.

### 3.6. Evidence-Based Medicine Research

Meta-analysis is used to compare and summarize the findings of research on the same scientific question based on statistical method. It is often used for quantitative combined analysis in systematic reviews.  Table 8 lists the top 20 cited systematic reviews and meta-analyses in FMT, and we can find FMT-relatedmeta-analyses mainly focused on several aspects, including CDI, IBD, UC, IBS, and so on.

### 3.7. Analysis of Keywords

#### 3.7.1. Common Keywords and Burst Keywords

**Figure 6:**
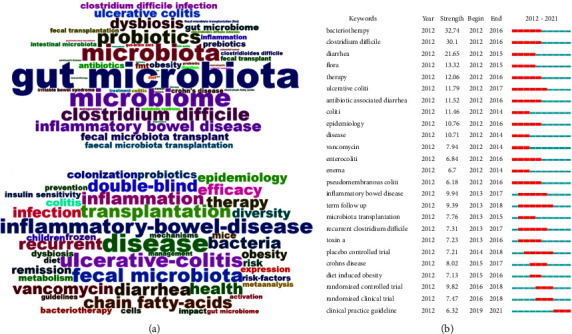
(a) Common FMT-related author keywords (above) and keywords plus (below). (b) The 25 bursts keywords (the years in green and red suggest that the keywords have less and greater effects, respectively).

A total of 8,116 keywords were extracted, including 4,001 author keywords and 4,165 keywords plus.  Figure 6(a) depicts the top 50 author keywords and keywords plus (excluding search terms). Among author's keywords, “*Clostridium difficile*,” “inflammatory bowel disease,” “ulcerative colitis,” “*Clostridium difficile* infection,” “obesity,” “antibiotics,” “prebiotics,” “inflammation,” “Crohn's disease,” “irritable bowel syndrome,” “gut-brain axis,” “metabolic syndrome,” “probiotic”, “cancer,” “short-chain fatty acids,” and “bile acids” were most used. Among keywords plus, “*Clostridium difficile* infection,” “inflammatory bowel disease,” “ulcerative-colitis,” “inflammation,” “double-blind,” “chain fatty acids,” “active ulcerative-colitis,” “Crohn's disease,” “obesity,” “metabolism,” “insulin sensitivity,” “irritable bowel syndrome,” and “meta-analysis” were most used.

The burst keywords can help us to know the evolutions and dynamics of hotspots, development trends, and frontier in a certain time.  Figure 6(b) depicts the top 25 burst keywords. As we can see, in the early years, FMT for CDI and antibiotic-associated diarrhea was the main focus. Subsequently, the focus was mainly on the effects of FMT in IBD especially UC, diet-induced obesity, and clinical practice guideline of FMT. Overall, FMT-related research had undergone the stages from infectious disease to noninfectious disease, from case reports to RCT studies, and from empirical application to clinical consensus issued.

#### 3.7.2. Cluster Analysis of Common Keywords

**Figure 7:**
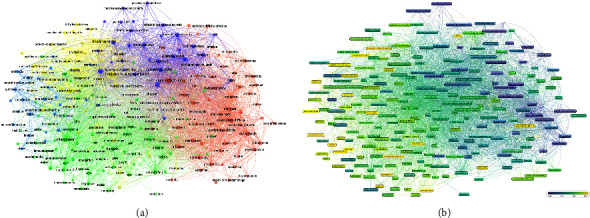
(a) Common keywords cluster analysis (various colors show various types, the circle's size manifests the keywords frequency, and line's width manifests the link intensity). (b) Common keyword evolution trend over time (the blue and yellow boxes show the earlier and latest keywords, respectively).

The cluster analysis is carried out based on cooccurrence keywords. This study uses hierarchical clustering to classify and merge the clustered keywords into a category, and proves the similarity of keywords in the field of FMT. We analyzed all the included keywords through VOSviewer, showing a network diagram of cooccurrence relationships.  Figure 7(a) shows the clustering analysis of common keywords (frequency set to 20), which was divided into five types.

 
*Cluster 1 (Red Topic).* This sort of keywords is principally related to the application of FMT in CDI. Major research topics include *Clostridium difficile* infection, antibiotic-associated diarrhea, diarrhea, clinical practice guidelines, risk factors, diagnosis, prevalence, prevention, and treatment.
 
*Cluster 2 (Green Topic)*. This sort of keywords is principally linked to the mechanisms of FMT and GM in health and disease, and involves many aspects such as immunity, metabolism, inflammation, expression, oxidative stress, and barrier function.
 
*Cluster 3 (Light Blue Topic).* This sort of keywords focused on FMT for neurological and psychiatric diseases. Major topics include Alzheimer's disease (AD), Parkinson's disease (PD), anxiety, depression, autism, stress, gut-brain axis, neuroinflammation, central nervous system, metabolome, and immune.
 
*Cluster 4 (Yellow Topic)*. This category is mainly linked to the application of FMT in metabolic syndrome. Major topics include obesity, insulin resistance, diabetes, fatty liver disease, cirrhosis, nonalcoholic steatohepatitis, Akkermansia muciniphila, chain fatty acids, bile acids, and glucagon-likepeptide-1. In addition, there was also the application of FMT in cancer, the main keywords include cancer, colorectal cancer, Fusobacterium nucleatum, immunotherapy, and chemotherapy.
 
*Cluster 5 (Deep Blue Topic)*. This category is mainly linked to the application of FMT in IBD and IBS. Major research topics include IBD, Crohn's disease, ulcerative colitis, IBS, butyrate-producing bacteria, and mucosa-associated microbiota.

#### 3.7.3. Trends Analysis of Common Keywords

Similar to concurrency graphs, overlay visual map in VOSviewer is a useful tool for forecasting future hotspots and trends in a variety of scientific domains. As seen in Figure 7(b), the purple circles indicate the earlier keywords and the yellow represent keywords that have appeared recently. From 2012 to 2021, there are relatively unbalanced trends in the five clusters, showing a tendency of diversified development. The trend in recent years (Figure 7(b)) shows that yellow nodes are mainly in the third and fourth cluster, and the main keywords include “Alzheimer's disease,” “Parkinson's disease,” “ brain axis,” “neuroinflammation,” “chemotherapy,” “immunotherapy,” “consensus statement,” and so on. These keywords mainly focus on neurological diseases and anticancer treatment.

## 4. Discussion

Bibliometric analysis can identify the characteristics of papers in specific research areas, visualize the collaboration network between countries, institutions, and authors, show the citations and milestone articles, with unique advantages, and are widely used in various research fields. As a treatment method that has been written into the guidelines, FMT for CDI treatment has been applied in some countries, and the scope of its clinical indications has a trend of further expansion. To gain a better overall understanding of FMT research, we conducted an analysis of global research papers in FMT from 2012 to 2021 to identify the status and trends of FMT research.

### 4.1. Characteristics of Papers in FMT

The Np in FMT research can show the evolution phases it had experienced. From the annual Np, the development of FMT research can be divided into three stages. FMT research was in the infancy stage before 2012 (total 25 papers in 2004–2011, but 26 papers in 2012). The poor progress of FMT research may be explained by the fact that GM research had just recently begun during this period, as seen by the introduction of the 2007 Human Microbiome Project. 2013–2016 was in the stable and slow growth stage, which may be related to the fact that FMT was officially written into the clinical guidelines of CDI, indicating that FMT is beginning to be recognized by most investigators. 2017–2021 was a high-yield period, and the Np in 2021 would reach its peak, indicating that FMT-related research is getting more and more attention, which may be due to the quick advancement of GM and FMT research, as well as rising researcher interest in FMT.

Few researchers know all relative journals in their field, and researchers struggle to choose the most appropriate journals to output their research. This can be drawn from journal metrics obtained from the bibliometric analysis. From the source of papers, we found that most of the FMT-related papers was mainly published in the specific journals, such as *Frontiers in Microbiology*, *Gut Microbes*, and *World Journal of Gastroenterology*, which are world class journals and have greater impact on FMT research and offer a publishing reference for FMT-related papers, and scholars may give priority to these journals. *Frontiers in Microbiology* ranked first in Np, TC, and H-index, it is a renowned microbiology journal which advances our grasp of the role of microbes in addressing global challenges such as healthcare. Highly cited papers were mainly published in very well-known medical journals, indicating that FMT-related research may represent medical cutting-edge research.

Most countries had participated in FMT research, of which the US and China had the highest Np, TC, and H-index, and were at the center of global cooperation, showing their important contributions to FMT research, which was linked to their strong interest and backing on the microflora projects. In research institutions, Harvard University, University of California System and Harvard Medical School from the United States, and Udice French Research Universities and Inserm from France, as the top universities and institutions in the world, had published most papers. From prolific authors, Nieuwdorp Max from the University of Amsterdam in the Netherlands had the highest Np, TC, and H-index, showing that his papers had a greater influence on FMT research, who may affect the focus and direction of FMT research. He mainly focused on MS [14], such as insulin sensitivity, and obesity. Allegretti, Jessica R., and Kassam Zain from the US mainly focused on FMT for UC [63], IBS, and CDI. Cammarota Giovanni, Ianiro Gianluca, and Gasbarrini Antonio from Italy focused on FMT in CDI, psychiatric disorders [64], and cancer treatment [65]. Khanna Sahil from Mayo Clinic, Khoruts Alexander from University of Minnesota, and Kelly Colleen R. from Brown University in the USA mainly focused on FMT in rCDI [66, 67]. Zhang Faming from Nanjing Medical University in China mainly focused on FMT in CD and UC, and some questionnaires and ethical issues on the perception of FMT among physicians and patient groups [68], and put forward the concept of washed microbiota transplantation (WMT) [69]. Notably, most of the top 10 authors participated in the formation of the FMT-related consensus [41, 70]. In order to know the latest research progress in FMT research, we should focus on their work and give their research a relative priority.

### 4.2. Current Frontiers and Trends in FMT

Common keywords are utilized to identify the hotspots, while the cluster analysis can locate the primary study materials under the hot topics. Common keywords and cluster analysis showed the primary status and hotspots in FMT, which mainly concentrated on the mechanism and treatment of FMT.

Currently, many studies have explored the clinical application of FMT, including the following aspects: (1) rCDI: The most efficient and well-researched indication for FMT to date is rCDI. Numerous studies had shown that FMT was established as a highly restorative treatment for rCDI [71]. Several meta-analyses had shown considerable promise for FMT in rCDI [29, 40, 67, 72–74]. The routes, infusions times, and fecal dose may affect the efficacy of FMT for rCDI [73]. Moreover, colonoscopy and the oral route were superior to stool enemas; FMT in relapsed CDI also was more effective than refractory CDI [67]. (2) IBD: IBD, especially UC, is another current hotspot in FMT. Two meta-analyses [75, 76] showed that FMT may be safe and efficient for IBD treatment. FMT was an efficient way for the treatment of CDI in IBD patients [77], FMT may be a novel therapeutic option for IBD. Some systematic reviews and meta-analyses suggested that FMT is a safe, well-tolerated, and effective treatment for certain diseases other than rCDI, with the most compelling evidence for active UC [78–80]. (3) IBS: Some studies have shown that FMT can help restore the GM and its function in IBS patients, and the richness and diversity of GM increased in IBS patients after FMT [8, 81]. Two meta-analyses showed that delivery of fresh or frozen donor feces may be beneficial for IBS [82, 83]. However, some studies also showed no disparity between FMT and control groups in RCTs in improvement or changes of the IBS symptoms and the living quality of patients, and FMT is considered ineffective for IBS [78, 83]. (4) MS: MS is a group of clinical syndromes characterized by central obesity, hyperglycemia, dyslipidemia, and hypertension, and insulin resistance serves as the common pathophysiological basis. Several studies had shown that FMT had advantages for MS, possibly improving insulin sensitivity by modifying the GM [13, 27, 84]. FMT may play a role in treating MS, but there is currently insufficient evidence to support its clinical practice [84]. (5) MD: Some papers showed that the pathogenesis of depression and anxiety disorders is closely linked to the changes of GM [15, 48, 49]. FMT may cure psychiatric disorders by adjusting the brain-gut-bacteria axis, providing new ideas for depression and anxiety disorders. FMT can effectively enhance psychiatric disorders in recipient animals. Preclinical and clinical studies suggested that reversing or alleviating dysbiosis appears to be a promising strategy for restoring behavioral disorders or achieving remission of psychiatric symptoms [64].

At present, the evidence-based medical research of FMT mainly focuses on CDI, IBD, and IBS (Table 8). Notably, FMT may play a role in IBS treatment, but there is currently insufficient witness to support its clinical application. For example, a 2020 meta-analysis showed that FMT markedly enhanced clinical remission rates in active UC, but there was no apparent change in IBS symptoms after FMT [78]. In addition, although this bibliometric study and some clinical trials had shown the potential therapeutic effect of FMT in some diseases such as MD and MS, there is still a lack of evidence-based medical studies to further verify its clinical efficacy and safety. With the expansion of FMT application, more randomized controlled clinical studies will be available for evidence-based medical analysis.

Many studies explored the therapeutic mechanism of FMT, which may achieve therapeutic purposes by realizing new GM-host interactions, but the concrete origin of interactions remains unclear. Specifically, the therapeutic effect of FMT is mainly mediated by the GM. Many studies show that there is a crosstalk among GM, metabolism, and immunity. GM dysbiosis can stimulate persistent inflammation, and affect the host immune system and metabolism. GM and its metabolites are critical for the development of host immunity, and in turn, host immunity also affects the GM [85]. The interaction of the GM-metabolome-immune network can be revealed through multiomics analysis, which is the current research focus [86]. The normal GM maintains the balance of local immune responses and barrier integrity in the gut by exposing LPS and metabolites such as short-chain fatty acids [87]. Reactive oxygen species also have a key role in inducing programmed cell death and many diseases, and oxidative stress can be better known and controlled by tracking oxidative stress levels in feces to find proinflammatory components [88].

There are also some yellow nodes in other clusters, but they are scattered. Among them, neurological diseases (ND) and antitumor chemotherapy and immunotherapy research have received more attention in recent years, which may suggest future research directions. The main keywords include AD, PD, chemotherapy, immunotherapy, and *Clostridium difficile* infection, showing that FMT in neurological diseases and antitumor drug treatment are the focus in the future. (1) Neurological diseases: GM plays a crucial role in the interplay between the gut and the brain, which could shape neurodevelopment, modulate neurotransmission, and influence behavior, thereby affecting ND [16]. FMT may be a promising therapeutic option for several ND. Recent publications have highlighted GM imbalances in the development and progression of ND, and GM-related interventions may be used to treat neurological disorders [16]. FMT derived from AD mouse can impair memory function and neurogenesis in mice [89]. FMT can protect rotenone-induced PD by inhibiting LPS-TLR4 signaling-mediated inflammation via the microbe-gut-brain axis [90]. But the current research is still mainly focused on basic research. (2) Cancer treatment: The GM may affect the efficacy and adverse effects of antitumor chemotherapeutics and immunotherapy. FMT is increasingly being studied to overcome cancer treatment resistance and side effects [91, 92]. An animal study showed FMT can prevent chemotherapy-induced intestinal mucositis in colorectal cancer [93]. Specific GM may contribute to chemotherapy-related side effects, and FMT can reverse chemotherapy-induced GM dysbiosis and side effects [94, 95]. For immunotherapy, in 2015, two papers in *Science* pointed out that the effect of CTLA-4 inhibitor depends on GM, and FMT can improve antitumor immune response and facilitate anti-PD-L1 efficacy [52, 53]. In 2018, two papers in *Science* found that the GM modulate antitumor response of the checkpoint blockade immunotherapy, and FMT can improve the effect of PD-1 inhibitors, which has important implications for research on the antitumor immunotherapy [50, 51]. In 2021, two trials published in *Science* showed that FMT from ICI responders can overcome the resistance to immunotherapy [11, 96]. Future efforts should focus on developing therapeutics targeting the GM. (3) Consensus and guidelines: Guidelines and consensus statements for FMT clinical practice can effectively standardize the diagnosis and treatment behavior of medical staff, improve the quality of medical services, and reduce medical costs. Several guidelines and consensus had been published regarding clinical FMT [41, 70, 97]. Fecal banks can give patients with consistent, timely, and equitable access to FMT, as well as traceable workflows to assure process safety and quality. An international consensus in 2019 provided detailed advice for FMT in clinical practice [70]. In addition, animal FMT has important implications for basic research, and the causal relationship between GM and disease models can be determined by performing FMT on animals such as mice [98]. A guideline reporting on animal FMT made detailed recommendations for FMT protocols from mice [99].

### 4.3. Limitations of the Study

The study also has several limitations. First, while the included papers adequately reflect the current state, we retrieved data only from the SCI-Expanded of WoSCC database. Second, bibliometric surveys of newly published high-quality articles will be ignored. Inherent biases such as bibliometrics against recently published papers may cause some significant papers to not be included in these analyses because it takes time to accumulate citations. Third, the impact of an article and the progress in a field cannot be known by the citations alone, nor should a low publication rate in a country imply a lower quality of scientific research. Therefore, there may be discrepancies between bibliometric analysis and real studies.

## 5. Conclusions

One of the study's main strengths is that by including all journals generated within the FMT study area, we generated a diverse top-cited composition of corresponding authors, journals, articles, countries, and institutions. Furthermore, research hotspots and trends connected to FMT are studied and projected using keyword analysis, providing study suggestions for future research. Further optimization of FMT methods, such as capsule preparations and frozen fecal bacteria, can reduce costs by reducing the number and frequency of donor screening, relieve patient discomfort during operation, and increase the acceptance of patients and medical staff, which has a good application prospect. Well-designed randomized controlled clinical studies and high-qualityevidence-based medical studies are needed to identify the best indications, maintenance methods, and transplantation pathways for FMT. The safety assessment of FMT is still in its infancy, and the consensus has not yet been formed, and more in-depth research is still needed. With the extensive attention of researchers and the advancement of technology, future research on FMT is likely to get rapid growth and previously unexpected applications, fecal therapy will continue to improve beyond “whole fecal” transplants. This study displayed the global research state and trends of FMT using bibliometrics and graphical analysis. It helps scholars in allied domains with a better grasp of the development and evolution process of FMT and provides a reference for the use of FMT in new disciplines by summarizing existing research hotspots and projecting future development trajectories.

## Figures and Tables

**Table 1:** The top 10 high-yield journals in FMT research.

No.	Journals	Np	TC	H-index	IF	Partitions	Countries
1	Frontiers in Microbiology	66	1900	22	6.064	Q1	Switzerland
2	Gut Microbes	62	1870	24	9.434	Q1	USA
3	World Journal of Gastroenterology	52	2431	25	5.374	Q2	USA
4	PLoS One	45	1641	24	3.752	Q2	USA
5	Microbiome	44	3361	26	16.837	Q1	UK
6	Scientific Reports	41	1510	19	4.996	Q2	UK
7	Frontiers in Immunology	40	924	14	8.786	Q1	Switzerland
8	Frontiers in Cellular and Infection Microbiology	38	647	12	6.073	Q2	Switzerland
9	Gastroenterology	33	6522	24	33.883	Q1	USA
10	Inflammatory Bowel Diseases	33	1430	17	7.290	Q1	USA

**Table 2:** The top 10 high-impact journals in FMT research.

No.	Journals	TC	Journals	H-index
1	Science	9744	Microbiome	26
2	Gastroenterology	6522	World Journal of Gastroenterology	25
3	Gut	4339	Gastroenterology	24
4	Microbiome	3361	Gut	24
5	Nature	3212	Gut microbes	24
6	American Journal of Gastroenterology	2932	PloS One	24
7	New England Journal of Medicine	2638	Alimentary Pharmacology & Therapeutics	23
8	Alimentary Pharmacology & Therapeutics	2567	Frontiers in Microbiology	22
9	World Journal of Gastroenterology	2431	Clinical Infectious Diseases	19
10	Cell	2136	Nature Communications	19

**Table 3:** The top 10 high-yield countries and agencies in FMT research.

No.	Countries	Np	Citation	H-index	Agencies	Np	Citation	H-index
1	USA	1050	56650	113	Harvard University (USA)	139	9212	48
2	China	755	19656	64	Udice French Research Universities (France)	110	10569	39
3	Canada	187	10933	54	University of California System (USA)	103	4410	33
4	Germany	171	8484	39	Inserm (France)	86	8720	34
5	Italy	170	8610	49	Harvard Medical School (USA)	73	3444	33
6	UK	157	7647	43	University of Amsterdam (Netherlands)	70	9124	38
7	France	150	13173	45	Assistance Publique Hopitaux Paris (France)	61	6600	28
8	Netherlands	132	15086	53	Nanjing Medical University (China)	61	1640	23
9	Australia	128	7859	42	Catholic University of the Sacred Heart (Italy)	57	3017	25
10	Spain	97	3496	27	Imperial College London (UK)	57	4208	25

**Table 4:** The top 10 high-yield authors in FMT research.

Rank	Authors	Np	TC	H-index	Affiliations	Countries
1	Nieuwdorp Max	44	7440	27	University of Amsterdam	Netherlands
2	Zhang Faming	44	1407	21	Nanjing Medical University	China
3	Khoruts Alexander	43	4513	27	University of Minnesota	USA
4	Allegretti Jessica	42	1575	19	Harvard Medical School	USA
5	Kassam Zain	41	3139	23	Finch Therapeutics	USA
6	Cammarota Giovanni	40	2357	21	Catholic University of the Sacred Heart	Italy
7	Ianiro Gianluca	40	2599	21	Catholic University of the Sacred Heart	Italy
8	Gasbarrini Antonio	38	2031	18	Catholic University of the Sacred Heart	Italy
9	Khanna Sahil	37	978	19	Mayo Clinic	USA
10	Kelly Colleen	35	3246	23	Brown University	USA

**Table 5:** The FMT-related classic papers in historical citation network.

No.	First author	Year	Journals	DOI	Document type	LCS	GCS
1	Vrieze	2012	Gastroenterology	10.1053/j.gastro.2012.06.031	Clinical study	326	1640
2	Brandt	2012	The American Journal of Gastroenterology	10.1038/ajg.2012.60	Clinical study	265	454
3	Hamilton	2012	The American Journal of Gastroenterology	10.1038/ajg.2011.482	Clinical study	295	454
4	Borody	2012	Nature Reviews Gastroenterology & Hepatology	10.1038/nrgastro.2011.244	Review	179	388
5	van Nood	2013	The New England Journal of Medicine	10.1056/NEJMoa1205037	Clinical RCT	895	2227
6	Kassam	2013	The American Journal of Gastroenterology	10.1038/ajg.2013.59	Review	342	579
7	Debast	2014	Clinical Microbiology and Infection	10.1111/1469–0691.12418	Review	241	807
8	Youngster	2014	JAMA	10.1001/jama.2014.13875	Clinical study	247	422
9	Kelly	2014	The American Journal of Gastroenterology	10.1038/ajg.2014.133	Clinical study	251	411
10	Youngster	2014	Clinical Infectious Diseases	10.1093/Cid/ciu135	Clinical study	197	300
11	Moayyedi	2015	Gastroenterology	10.1053/j.gastro.2015.04.001	Clinical RCT	435	816
12	Rossen	2015	Gastroenterology	10.1053/j.gastro.2015.03.045	Clinical study	314	529
13	Cammarota	2015	Alimentary Pharmacology & Therapeutics	10.1111/apt.13144	Clinical RCT	251	358
14	Kelly	2015	Gastroenterology	10.1053/j.gastro.2015.05.008	Review	176	347
15	Lee	2016	JAMA	10.1001/jama.2015.18098	Clinical RCT	262	390
16	Kelly	2016	Annals of Internal Medicine	10.7326/M16-0271	Clinical study	206	343
17	Paramsothy	2017	Lancet	10.1016/S0140-6736(17)30182-4	Clinical RCT	306	611
18	Cammarota	2017	Gut	10.1136/Gutjnl-2016-313017	Clinical study	288	497
19	Kao	2017	JAMA	10.1001/jama.2017.17077	Clinical RCT	174	283
20	Quraishi	2017	Alimentary Pharmacology & Therapeutics	10.1111/apt.14201	Review	172	282

**Table 6:** The top 20 high-cited FMT-related articles.

No.	DOI	First author	Year	Journals	IF	JCR	TC
1	10.1056/NEJMoa1205037	van Nood	2013	The New England Journal of Medicine	176.079	Q1	2227
2	10.1126/science.aan3706	Routy and Bertrand	2018	Science	63.714	Q1	2172
3	10.1126/science.aan4236	Gopalakrishnan	2018	Science	63.714	Q1	1868
4	10.1126/science.aac4255	Sivan and Ayelet	2015	Science	63.714	Q1	1771
5	10.1053/j.gastro.2012.06.031	Vrieze and Anne	2012	Gastroenterology	33.883	Q1	1640
6	10.1126/science.aad1329	Vetizou	2015	Science	63.714	Q1	1630
7	10.1126/science.1233521	Markle and Janet	2013	Science	63.714	Q1	1094
8	10.1038/nature14232	Chassaing and Benoit	2015	Nature	69.504	Q1	938
9	10.1038/nature13793	Suez and Jotham	2014	Nature	69.504	Q1	916
10	10.1038/mp.2016.44	Zheng	2016	Molecular Psychiatry	13.437	Q1	845
11	10.1053/j.gastro.2015.04.001	Moayyedi and Paul	2015	Gastroenterology	33.883	Q1	816
12	10.1038/nature11228	Hashimoto and Tatsuo	2012	Nature	69.504	Q1	692
13	10.1016/j.jpsychires.2016.07.019	Kelly and John	2016	Journal of Psychiatric Research	5.250	Q2	668
14	10.1186/s40168-016-0222-x	Li Jing	2017	Microbiome	16.837	Q1	650
15	10.1016/j.cell.2014.09.048	Thaiss Christoph	2014	Cell	66.850	Q1	635
16	10.1016/S0140-6736(17)30182-4	Paramsothy Sudarshan	2017	Lancet	202.731	Q1	611
17	10.1186/s40168-016-0225-7	Kassam Zain	2017	Microbiome	16.837	Q1	554
18	10.1053/j.gastro.2015.03.045	Rossen Noortje	2015	Gastroenterology	33.883	Q1	529
19	10.1016/j.cell.2015.10.048	Levy Maayan	2015	Cell	66.850	Q1	505
20	10.1038/nm.4068	Benakis Corinne	2016	Nature Medicine	87.241	Q1	474

**Table 7:** The high-cited references related to FMT research.

No.	First author	Years	Pathway	Indications	Case load	Journals	Citations
1	Eiseman	1958	Enema	Pseudomembranous enterocolitis	4	Surgery	305
2	Bowden	1981	Small intestinal tube	Pseudomembranous enterocolitis	16	Am surgeon	35
3	Schwan	1983	Enema	Pseudomembranous enterocolitis	1	Lancet	64
4	Bennet	1989	Enema	Ulcerative colitis	1	Lancet	103
5	Borody	1989	Colonoscopy	IBD and IBS	55	Medical Journal of Australia	98
6	Tvede	1989	Enema	rCDI-related diarrhea	1	Lancet	84
7	Persky	2000	Colonoscope	rCDI-related diarrhea	1	The American Journal of Gastroenterology	53
8	Aas	2003	Nasogastric tube	rCDI-related diarrhea	18	Clinical Infectious Diseases	140
9	Borody	2003	Enema	Ulcerative colitis	6	Journal of Clinical Gastroenterology	119

**Table 8:** The top 20 high-cited FMT-related systematic reviews and meta-analyses.

No.	Applications	DOI	First author	Year	Journals	IF	JCR	TC
1	*Clostridium difficile* infection	10.1038/ajg.2013.59	Kassam	2013	The American Journal of Gastroenterology	12.045	Q1	579
2	Inflammatory bowel disease	10.1016/j.crohns.2014.08.006	Colman	2014	Journal of Crohn's and Colitis	10.020	Q1	283
3	Recurrent and refractory CDI	10.1111/apt.14201	Quraishi	2017	Alimentary Pharmacology & Therapeutics	9.524	Q1	282
4	Inflammatory bowel disease	10.1093/ecco-jcc/jjx063	Paramsothy	2017	Journal of Crohn's and Colitis	10.020	Q1	225
5	Active ulcerative colitis	10.1111/apt.14173	Costello	2017	Alimentary Pharmacology & Therapeutics	9.524	Q1	150
6	Active ulcerative colitis	10.1097/MIB.0000000000001228	Narula	2017	Inflammatory Bowel Disease	7.290	Q1	111
7	*Clostridium difficile* infection	10.1111/apt.13492	Li	2016	Alimentary Pharmacology & Therapeutics	9.524	Q1	97
8	*Clostridium difficile* infection	10.1177/2050640618780762	Ianiro	2018	United European Gastroenterology Journal	6.866	Q1	92
9	Irritable bowel syndrome	10.1111/apt.15330	Ianiro	2019	Alimentary Pharmacology & Therapeutics	9.524	Q1	79
10	CDI-associated diarrhea	10.5694/mja17.00295	Moayyedi	2017	Medical Journal of Australia	12.776	Q1	78
11	Recurrent CDI	10.1093/Cid/ciy721	Tariq	2019	Clinical Infectious Diseases	20.999	Q1	74
12	Irritable bowel syndrome	10.14309/ajg.0000000000000198	Xu	2019	The American Journal of Gastroenterology	12.045	Q1	72
13	Inflammatory bowel disease	10.1080/19490976.2017.1353848	Qazi	2017	Gut Microbes	9.434	Q1	72
14	Recurrent CDI	10.1371/journal.pone.0210016	Hui	2019	PLoS One	3.752	Q2	58
15	Total outcomes in FMT	10.1111/apt.15116	Lai	2019	Alimentary Pharmacology & Therapeutics	9.524	Q1	57
16	Chronic refractory pouchitis	10.1111/apt.13905	Segal	2017	Alimentary Pharmacology & Therapeutics	9.524	Q1	56
17	Ulcerative colitis	10.1371/journal.pone.0157259	Shi	2016	PLoS One	3.752	Q2	46
18	Inflammatory bowel disease	10.1155/2018/8941340	Fang	2018	BioMed Research International	3.246	Q3	44
19	Recurrent CDI	10.1016/j.eclinm.2020.100642	Baunwall	2020	eClinicalMedicine	17.033	Q1	34
20	CDI in IBD patients	10.1093/ecco-jcc/jjy031	Chen	2018	Journal of Crohn's and Colitis	10.020	Q1	30

## Data Availability

The Web of Science database contains the original data that were used in the study. The associated authors can be contacted for more information. The search link was as follows: https://www.webofscience.com/wos/woscc/summary/5c068744-8c95-48a4-858f-14d0d436fe16-483dcde5/times-cited-descending/1.
